# East Asian herbal medicine for cancer pain: A protocol for systematic review and meta-analysis with using association rule analysis to identify core herb pattern

**DOI:** 10.1097/MD.0000000000027699

**Published:** 2021-11-12

**Authors:** Hee-Geun Jo, Donghun Lee

**Affiliations:** a Chung-Yeon Central Institute, 64, Sangmujungang-ro, Seo-gu, Gwangju, Republic of Korea; b Department of Herbal Pharmacology, College of Korean Medicine, Gachon University, 1342 Seongnamdae-ro, Sujeong-gu, Seongnam, Republic of Korea.

**Keywords:** association rule mining, cancer pain, complementary and alternative medicine, East Asian herbal medicine, meta-analysis, systematic review

## Abstract

**Background:**

Cancer pain is an important factor in cancer management that affects a patient's quality of life and survival-related outcomes. The aim of this review is to systematically evaluate the efficacy and safety of oral administration East Asian herbal medicine (EAHM) for primary cancer pain, and to explore core herb patterns based on collected data.

**Methods:**

A comprehensive literature search will be conducted in 10 electronic databases including PubMed, Cochrane Library, Cumulative Index to Nursing & Allied Health Literature, EMBASE, Korean Studies Information Service System, Research Information Service System Oriental Medicine Advanced Searching Integrated System, Korea Citation Index, Chinese National Knowledge Infrastructure Database (CNKI), CiNii for randomized controlled trials from their inception until August 19, 2021. Statistical analysis will be performed in the software R version 4.1.1. and R studio program using the default settings of the ‘meta’ package. When heterogeneity in studies is detected, the cause will be identified through meta regression and subgroup analysis. Methodological quality will be assessed independently using the revised tool for risk of bias in randomized trials (Rob 2.0).

**Results:**

This study will provide more comprehensive and specific evidence of EAHM for cancer pain management.

**Conclusions:**

Based on the results of this review, it is expected that the efficacy and safety of East Asian herbal medicine for cancer pain may be confirmed. In addition, it will be possible to derivation of a core herb pattern related to this research topic through additional association rule mining analysis.

## 1 Introduction

Pain is an important factor influencing clinical outcome in the medical management of cancer. Recent literature on the prevalence of cancer pain reports that not only 60% of patients with advanced or metastatic cancer complain of pain, but also moderate to severe pain is observed in more than one-third of the patients.^[1,2]^ Although clinicians’ awareness of cancer pain is gradually improving, there is still a report that about one-third of cancer survivors do not have access to proper management.^[3]^ In addition to this, a significant number of patients still suffer from pain after completing curative treatment.^[1]^ Therefore, preparing a more effective and safer treatment strategy for cancer pain is an urgent task in clinical research above all else.

Currently, the WHO Analgesic ladder is widely used as a framework for managing cancer pain. According to this recommendation, drugs ranging from over-the-counter analgesics to strong opioids can be administered sequentially as the severity of pain increases.^[4]^ However, a large number of patients complain of severe pain that does not respond to treatment even after receiving opioids.^[5]^ Meanwhile, concerns of medical consumers about opioids administration due to the continuous increase in accidental prescription opioid overdose or patients’ financial problems are also pointed out as important barriers.^[6,7]^

In this context, studies on various integrative therapies that can be used as therapeutic alternatives or to increase patient compliance with first-line pharmacologic treatment for cancer pain are being actively conducted recently.^[8]^ In particular, herbal medicine has been widely used as an intervention to relieve pain caused by various diseases for a long time in East Asian countries such as Korea, Taiwan, Japan, and China.^[9–12]^ Recently, a number of clinical and experimental studies on various problems caused by cancer have been reported based on the scientific method-based approach to East Asian herbal medicine (EAHM).^[13–15]^

Therefore, we set the following research objectives to provide meaningful evidence to clinicians by comprehensively reviewing the efficacy and safety of EAHM for cancer pain and to explore useful hypotheses for drug discovery. Systematic literature review on the efficacy and safety of overall oral EAHM focusing on improvement of pain intensity and response rate of cancer pain excluding secondary pain caused by anti-cancer treatment. Apriori algorithm-based association rule mining is performed on the herb data collected in this review to discover core herb pattern.

## 2 Methods

The present review will be conducted in accordance with the preferred reporting items for systematic reviews and meta-analysis (PRISMA) 2020 statement.^[16]^ The protocol of this systematic review was prepared according to preferred reporting items for systematic review and meta-analysis protocols (PRISMA-P) 2015,^[17]^ and was pre-registered in PROSPERO (Registration Number: CRD42021265804. Available from: https://www.crd.york.ac.uk/prospero/display_record.php?ID=CRD42021265804).

### 2.1 Search strategy

Randomized controlled trials (RCTs) that evaluated the efficacy of EAHM for cancer pain will be searched in the following 10 electronic databases from their inception until August 19, 2021: 4 English databases (PubMed, Cochrane Library, Cumulative Index to Nursing & Allied Health Literature, Excerpta Medica database), 4 Korean databases (Korean Studies Information Service System, Research Information Service System, Oriental Medicine Advanced Searching Integrated System [OASIS], Korea Citation Index), 1 Chinese databases (Chinese National Knowledge Infrastructure Database), 1 Japanese database (Citation Information by NII). The following Boolean format will be used for the search: (Pain[MeSH] OR Pain∗[TIAB] OR analgesia OR analges∗ OR nocicept∗ OR neuroapth∗) AND (“Cancer pain”[TIAB] OR “Cancer patient”[TIAB] OR “Cancer patients”[TIAB] OR Neoplasms[MeSH] OR Neoplasms∗[TI] OR Cancer∗[TI] OR Tumor∗[MeSH] OR Tumor∗[TI] OR Carcinoma[MeSH] OR Carcinoma∗[TI] OR Adenocarcinoma[MeSH] OR Adenocarcinoma∗[TI] OR adenomatous[TI] OR Lymphoma[MeSH] OR lymphom∗[TI] OR lymphedema∗[TI] OR Sarcoma[MeSH] OR Sarcoma∗[TI] OR “Antineoplastic agents”[MeSH] OR antineoplas∗[TI] OR ((adenom∗[TI] OR adenopath∗[TI]) AND malignant∗[TI]))) AND (“Plants, Medicinal”[MeSH] OR “Drugs, Chinese Herbal”[MeSH] OR “Medicine, Chinese Traditional”[MeSH] OR “Medicine, Kampo”[MeSH] OR “Medicine, Korean Traditional”[MeSH] OR “Herbal Medicine”[MeSH] OR “Prescription Drugs”[MeSH] OR “traditional Korean medicine”[TIAB] OR “traditional Chinese medicine”[TIAB] OR “traditional oriental medicine”[TIAB] OR “Kampo medicine”[Title/abstract] OR herb∗[TIAB] OR decoction∗[TIAB] OR botanic∗[TIAB]).

### 2.2 Study selection

#### 2.2.1 Type of studies

**Figure 1:**
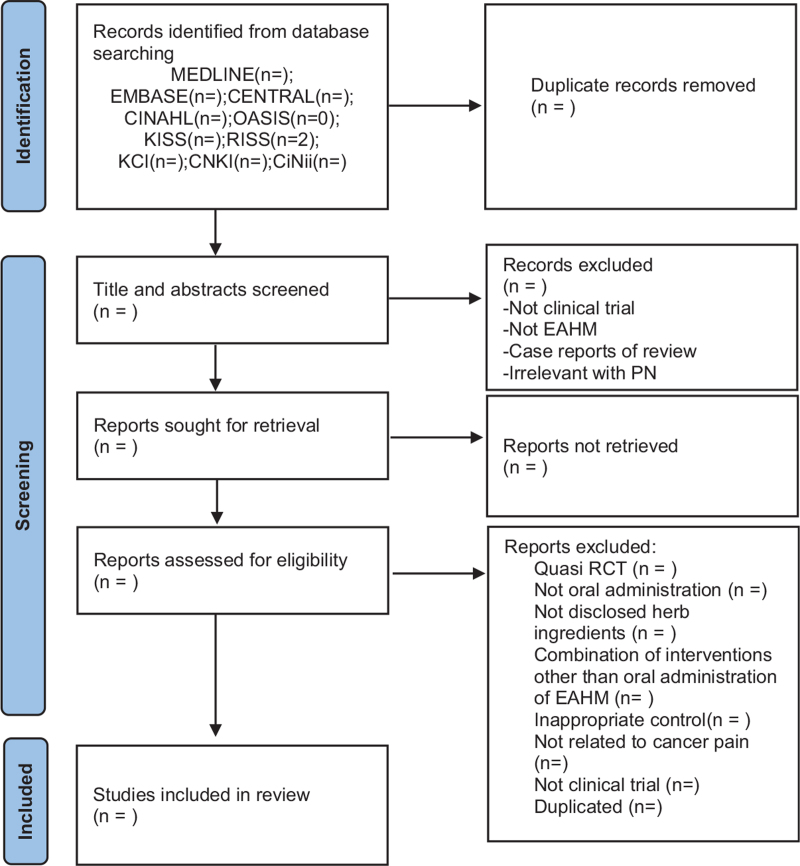
PRISMA 2020 flow diagram. PRISMA = preferred reporting items for systematic review and meta-analysis.

Only RCTs evaluating the efficacy and safety of oral administration of EAHM for cancer pain will be included. There will be no restrictions on language and publication time. Some studies will be excluded if they met the following criteria: not RCT or quasi RCT; the control group is not used or is inappropriate; unrelated to cancer pain; animal experiments; case reports or review; and not published in scientific peer-reviewed journals, including postgraduate theses or dissertations. A PRISMA 2020 flow chart will be produced to show the number of articles identified, screened, included, and excluded (shown in Fig. 1).

#### 2.2.2 Type of participants

Trials will be considered eligible for inclusion if they were conducted in patients with cancer pain, with no restriction on age, sex, or race. However, studies in which patients’ secondary cancer related pain induced by other antitumor treatments such as chemotherapy or surgery were enrolled will be excluded because this review set primary cancer pain as the target disease.

#### 2.2.3 Type of interventions

RCTs that compared EAHM as the active intervention in the treatment group versus placebo or conventional medicine (CM) in the control group will be included. RCTs that tested EAHM combined CM versus CM alone will be also considered. All forms of EAHM such as decoction, granule, capsule for the management of cancer pain will be included. There will be no restrictions on the dose and duration of treatment for EAHM, but the mode of delivery will be limited to oral intake. Studies in which East Asian medical interventions such as acupuncture, massage, or non-drug therapy were only combined in the treatment group will be excluded. Studies in which the comparators included other EAHMs will be excluded. In addition, studies that could not confirm the composition of individual ingredients and herbs of the utilized EAHM prescription will be also excluded.

#### 2.2.4 Type of outcome measures

The primary outcome for cancer pain patients will be the response rate of remission for each group measured using the verbal rating scale (VRS), numerical rating scale (NRS), and visual analogue scale (VAS). In addition, individual continuous pain intensity outcomes such as NRS and VAS will be also adopted as primary outcomes. It will be used as a secondary outcome that closely reflects outcomes in patients with cancer pain, such as duration of pain relief, performance status, and opioid use. Meanwhile, to evaluate the safety of the intervention for cancer patients, the incidence of adverse events (AE) will also be included as a secondary endpoint.

#### 2.2.5 Data extraction

Titles and abstracts of potentially eligible studies will be independently screened by 2 investigators according to the search strategy mentioned above. For the selected studies, 2 reviewers will independently collect the following information from the included studies: title, first author's name, year of publication, sample size, age of participants, sex distribution, study design, cancer type, treatment and interventions for controls, duration of treatment, outcome measures, reported adverse events, and dosages of EAHMs. Any discrepancies will be resolved through discussion among researchers.

#### 2.2.6 Methodological quality assessment

The methodological quality of each included study will be independently evaluated by 2 investigators according to Rob 2.0, a modified tool for risk of bias (RoB) in randomized trials.^[18]^ Rob 2.0 consists of 5 domains: bias arising from the randomization process, bias deviating from the intended intervention, bias due to omission of outcome data, and bias in the selection of reported outcomes. Methodological quality is “high risk of bias.” It will be assessed on 3 levels of “low risk of bias” and “some concerns,” and discrepancies between raters will be resolved through discussion.

#### 2.2.7 Statistical analysis

##### 2.2.7.1 Evidence synthesis

Evidence synthesis of included studies with available data will be performed by calculating the effect size and 95% CI using only the random effect model. Heterogeneity will be considered statistically significant when the *P*-value based on the chi-squared test is <.10 or *I*^2^ is ≥50%. Two-sided *P* < .05 will be considered statistically significant. Statistical synthesis of individual research results will be performed in the software R version 4.1.1 and R studio program (Version 1.4.1106, Integrated Development for R. RStudio, PBC, Boston, MA) using the default settings of the “meta” package.^[19]^ In this review, in order to effectively reveal the exact value of the effect size without relying only on the *P* < .05 significance threshold in the interpretation of the primary outcome synthesis result, a drapery plot will be additionally illustrated along with the forest plot.^[20]^ Summary relative risk (RR) and 95% confidence interval (CI) will be calculated for response rate. Standardized mean difference (SMD) and 95% CIs will be calculated for continuous pain intensity and duration of pain relief. Mean difference (MD) and 95% CIs will be calculated for opioid usage and performance status. AEs will be calculated using the odds ratio because the probability of an event is significantly lower than that of other outcomes and it is necessary to estimate a causal relationship. The results of individual studies using outcomes for which synthesis is inadequate will be described in the text. If heterogeneity is detected in the synthesized results through meta-analysis, meta regression analysis and subgroup analysis will be performed to determine the cause. In order to distinguish publication bias, a contour-enhanced funnel plot will be used for the outcome that included the most studies.^[21]^ For the asymmetry on the visually confirmed funnel plot, Egger test^[22]^ and Begg test^[23]^ will be additionally performed to specifically confirm the existence of publication bias.

##### 2.2.7.2 Association rule mining

By analyzing the constituent herb data of EAHM collected from the included study, the potential association rules of core herb combinations will be explored. Before proceeding with this analysis, preliminary information for data mining will be extracted by first analyzing the frequency of individual herbs. The R studio program (Version 1.4.1106, Integrated Development for R. RStudio, PBC, Boston, MA) will be used for the Apriori association rule analysis and plot production. A data fit will be done by using “arules” package in R studio.^[24]^ The function of the R package “arulesViz” will be applied to generate graphical presentations according to the results.^[25]^ Mining of frequent hub item sets and association rules will be performed according to the Apriori algorithm method for discovering meaningful relationships between variables in a large database.^[26]^ Through this, it is possible to identify the elements composing the data and the relationship between the elements, and it is being used in various types of medical research aimed at predicting the characteristics of interventions.^[27–29]^

In the Apriori algorithm, support, confidence, and lift are the main metrics for measuring association. A rule is defined as an expression *X*⇒*Y* where *X*, *Y* ⊆ *I* and *X*∩*Y* = ∅. The herb *X* and herb *Y* are called antecedent (left hand side, LHS) and consequent (right hand side, RHS) of the rules. Association rules are rules which surpass researcher-specified minimum support and minimum confidence thresholds. The support, supp(*X*), of an itemset *X* is a measure of importance defined as the proportion of transactions in the dataset which contain the itemset. The confidence of a rule is defined as conf(*X*⇒*Y*) = supp(*X*∪*Y*)/supp(*X*), measuring how likely it is to see herb *Y* in a transaction containing herb *X*. An association rule *X*⇒*Y* needs to satisfy supp(*X*∪*Y*) ≥ *σ* and conf(*X*⇒*Y*) ≥ *δ*, where *σ* and *δ* are the minimum support and minimum confidence, respectively. Confidence can be interpreted as an estimate of the probability *P*(*Y*|*X*), the probability of finding the RHS of the rule in transactions under the condition these transactions also contain the LHS. Lift of a rule is defined as lift(*X*⇒*Y*) = (supp(*X*∪*Y*)/supp(*X*)). Support is a measure to evaluate the usefulness of the association rule and is the proportion of prescriptions containing a specific herb combination pattern in the total EAHM prescription. When the confidence is close to 1, herb A and herb B are irrelevant because they are close to independence in probability. Meanwhile, if the lift value is large, the correlation is interpreted as strong. In this review, the association rules will be identified based on the minimum values for support and confidence being 20% and 80%, respectively. And it will be deriving the core herb pattern and its constituent herbs that show the most obvious association among them.

#### 2.2.8 Quality of evidence according to outcome measurements

The overall quality of evidence for each outcome will be evaluated according to the grading of recommendations assessment, development, and evaluation pro.^[30]^ The grading quality of evidence and strength of recommendations in clinical practice guidelines assessment evaluates the overall quality of evidence in four levels: very low, low, moderate, and high. The level of evidence is lowered according to factors such as risk of bias, inconsistency, indirectness, imprecision, and publication bias, respectively.

## 3 Amendments

If there is a significant modification or change of this protocol, the details and date of all amendments will be described in the final report.

## 4 Ethics and dissemination

In the process of implementing this systematic review, personal information will not be disclosed or published. This review will not infringe the rights of the subjects. Since it is not a clinical study that directly recruited subjects, ethical approval is not possible. The results of this study will be reported in a peer-reviewed scientific journal.

## 5 Discussion

This systematic review will provide comprehensive information on the efficacy and safety of EAHM for cancer pain. Several systematic reviews have already been reported to investigate the palliative effects of herbal medicine for cancer pain.^[31–35]^ Nevertheless, evidence related to the efficacy of EAHM for cancer pain in general is still insufficient. More RCTs have been additionally conducted thanks to the quantitative expansion of EAHM-related scientific research in recent years. Thus, systematic reviews that reflect these results need to be continued. On the other hand, prior reviews for EAHM comprehensively deal with several types of EAHM formulas including multiple herbal ingredients, unlike reviews on CM with a single dose and composition. For this reason, it is difficult to identify which of the many herb-related information reflected in the review is useful information.

In such a situation, the evidence according to this review will provide more comprehensive and specific information to both clinicians who manage cancer pain. In addition, it is expected that the derivation of core herb pattern using data mining techniques can be used as a hypothesis worthy of follow-up research in the development of new drugs related to the subject.

## Acknowledgment

Funders did not contribute to the writing of this protocol.

## Author contributions

**Conceptualization:** Hee-Geun Jo, Donghun Lee.

**Data curation:** Hee-Geun Jo, Donghun Lee.

**Formal analysis:** Hee-Geun Jo.

**Funding acquisition:** Donghun Lee.

## References

[1] van den Beuken-van Everdingen MHJ de Rijke JM Kessels AG Schouten HC van Kleef M Patijn J. Prevalence of pain in patients with cancer: a systematic review of the past 40 years. Ann Oncol 2007;18:1437–49. 17355955 10.1093/annonc/mdm056

[2] van den Beuken-van Everdingen MHJ Hochstenbach LMJ Joosten EAJ Tjan-Heijnen VCG Janssen DJA. Update on prevalence of pain in patients with cancer: systematic review and meta-analysis. J Pain Symptom Manage 2016;51:1070.e9–90.e9. 27112310 10.1016/j.jpainsymman.2015.12.340

[3] Greco MT Roberto A Corli O. Quality of cancer pain management: an update of a systematic review of undertreatment of patients with cancer. J Clin Oncol 2014;32:4149–54. 25403222 10.1200/JCO.2014.56.0383

[4] Vargas-Schaffer G. Is the WHO analgesic ladder still valid? Twenty-four years of experience. Can Fam Physician Med Fam Can 2010;56:514–7. e202–e205. PMC2902929 20547511

[5] Anderson KO Mendoza TR Valero V. Minority cancer patients and their providers: pain management attitudes and practice. Cancer 2000;88:1929–38. 10760771

[6] Calcaterra S Glanz J Binswanger IA. National trends in pharmaceutical opioid related overdose deaths compared to other substance related overdose deaths: 1999–2009. Drug Alcohol Depend 2013;131:263–70. 23294765 10.1016/j.drugalcdep.2012.11.018 PMC3935414

[7] Kwon JH. Overcoming barriers in cancer pain management. J Clin Oncol 2014;32:1727–33. 24799490 10.1200/JCO.2013.52.4827

[8] Deng G. Integrative medicine therapies for pain management in cancer patients. Cancer J Sudbury Mass 2019;25:343–8. 10.1097/PPO.0000000000000399 PMC6777858 31567462

[9] Chen H-Y Lin Y-H Su IH Chen Y-C Yang S-H Chen J-L. Investigation on Chinese herbal medicine for primary dysmenorrhea: implication from a nationwide prescription database in Taiwan. Complement Ther Med 2014;22:116–25. 24559826 10.1016/j.ctim.2013.11.012

[10] Lin P-H Lin S-K Hsu R-J Cheng K-C Liu J-M. The Use and the prescription pattern of traditional chinese medicine among urolithiasis patients in Taiwan: a population-based study. J Altern Complement Med 2016;22:88–95. 26359806 10.1089/acm.2015.0116

[11] Arai Y-C Makino I Ikemoto T Saisu H Terajima Y Owari K. Kampo for the treatment of pain in japan: a review. Pain Ther 2020;9:161–70. 32157597 10.1007/s40122-020-00160-w PMC7203354

[12] Wang C Meng Q. Global research trends of herbal medicine for pain in three decades (1990–2019): a bibliometric analysis. J Pain Res 2021;14:1611–26. 34113168 10.2147/JPR.S311311 PMC8187106

[13] Lin T-H Chen S-I Su Y-C Lin M-C Lin H-J Huang S-T. Conventional Western treatment combined with chinese herbal medicine alleviates the progressive risk of lung cancer in patients with chronic obstructive pulmonary disease: a nationwide retrospective cohort study. Front Pharmacol 2019;10:987. 31572178 10.3389/fphar.2019.00987 PMC6753872

[14] Tsai F-J Liu X Chen C-J. Chinese herbal medicine therapy and the risk of overall mortality for patients with liver cancer who underwent surgical resection in Taiwan. Complement Ther Med 2019;47:102213. 31780007 10.1016/j.ctim.2019.102213

[15] Kim D Park M Haleem I. Natural product Ginsenoside 20(S)-25-methoxyl-dammarane-3β, 12β, 20-triol in cancer treatment: a review of the pharmacological mechanisms and pharmacokinetics. Front Pharmacol 2020;11:521. 32425780 10.3389/fphar.2020.00521 PMC7212460

[16] Page MJ McKenzie JE Bossuyt PM. The PRISMA 2020 statement: an updated guideline for reporting systematic reviews. BMJ 2021;372:n71. 33782057 10.1136/bmj.n71 PMC8005924

[17] Shamseer L Moher D Clarke M. Preferred reporting items for systematic review and meta-analysis protocols (PRISMA-P) 2015: elaboration and explanation. BMJ 2015;350:g7647. 25555855 10.1136/bmj.g7647

[18] Sterne JAC Savović J Page MJ. RoB 2: a revised tool for assessing risk of bias in randomised trials. BMJ 2019;366:14898. 10.1136/bmj.l4898 31462531

[19] Lortie CJ Filazzola A. A contrast of meta and metafor packages for meta-analyses in R. Ecol Evol 2020;10:10916–21. 33144936 10.1002/ece3.6747 PMC7593135

[20] Rücker G Schwarzer G. Beyond the forest plot: the drapery plot. Res Synth Methods 2021;12:13–9. 32336044 10.1002/jrsm.1410

[21] Peters JL Sutton AJ Jones DR Abrams KR Rushton L. Contour-enhanced meta-analysis funnel plots help distinguish publication bias from other causes of asymmetry. J Clin Epidemiol 2008;61:991–6. 18538991 10.1016/j.jclinepi.2007.11.010

[22] Egger M Davey Smith G Schneider M Minder C. Bias in meta-analysis detected by a simple, graphical test. BMJ 1997;315:629–34. 9310563 10.1136/bmj.315.7109.629 PMC2127453

[23] Begg CB Mazumdar M. Operating characteristics of a rank correlation test for publication bias. Biometrics 1994;50:1088–101. 7786990

[24] Hahsler M Grün B Hornik K. arules - a computational environment for mining association rules and frequent item sets. J Stat Softw 2005;14:01–25.

[25] Hahsler M. arulesViz: interactive visualization of association rules with R. R J 2017;9:163–75.

[26] Agrawal R Imieliński T Swami A. Mining association rules between sets of items in large databases. Proc 1993 ACM SIGMOD Int Conf Manag Data 1993;207–16.

[27] Leem J Jung W Kim Y Kim B Kim K. Exploring the combination and modular characteristics of herbs for alopecia treatment in traditional Chinese medicine: an association rule mining and network analysis study. BMC Complement Altern Med 2018;18:204. 29973199 10.1186/s12906-018-2269-7 PMC6030800

[28] Hsieh P-C Cheng C-F Wu C-W. Combination of acupoints in treating patients with chronic obstructive pulmonary disease: an apriori algorithm-based association rule analysis. Evid-Based Complement Altern Med 2020;2020:8165296. 10.1155/2020/8165296 PMC7256717 32595739

[29] Lin Y-H Wu H-C Hsieh P-C Tzeng I-S Wu S-Y Kuo C-Y. An association rule analysis of combined acupoints for the treatment of patients with dry eye disease. Complement Med Res 2021;28:317–24. 33333519 10.1159/000512674

[30] Guyatt GH Oxman AD Vist GE. GRADE: an emerging consensus on rating quality of evidence and strength of recommendations. BMJ 2008;336:924–6. 18436948 10.1136/bmj.39489.470347.AD PMC2335261

[31] Wang S-J Xu J Gong D-D Man C-F Fan Y. Meta-analysis of oral Chinese herbal medicine as an adjuvant treatment in relieving pain secondary to bone metastases. Chin J Integr Med 2013;Epub ahead of print. 10.1007/s11655-013-1553-0 24126977

[32] Wang N Xu L Wang J-S. Traditional Chinese medicine on treating pain caused by prostate cancer: a systematic review and meta-analysis. Medicine (Baltimore) 2019;98:e17624. 31689770 10.1097/MD.0000000000017624 PMC6946298

[33] Wang Y-H Chang J-Y Feng L. Effect of oral Chinese medicine combined with Western medicine on cancer pain: a meta-analysis. Chin J Integr Med 2021;27:713–20. 32820454 10.1007/s11655-020-3423-x

[34] Lee J-W Lee WB Kim W Min B-I Lee H Cho S-H. Traditional herbal medicine for cancer pain: a systematic review and meta-analysis. Complement Ther Med 2015;23:265–74. 25847565 10.1016/j.ctim.2015.02.003

[35] Lee SM Choi HC Hyun MK. An overview of systematic reviews: complementary therapies for cancer patients. Integr Cancer Ther 2019;18:1534735419890029. 31876212 10.1177/1534735419890029 PMC6933541

